# Balloon pulmonary valvotomy as interim palliation for symptomatic young infants with tetralogy of Fallot

**DOI:** 10.4103/0974-2069.41049

**Published:** 2008

**Authors:** K.S. Remadevi, Balu Vaidyanathan, Edwin Francis, B.R.J. Kannan, Raman Krishna Kumar

**Affiliations:** Division of Pediatric Cardiology, Amrita Institute of Medical Sciences and Research Center, Kochi, India

**Keywords:** Balloon valvuloplasty, congenital heart disease, cyanosis

## Abstract

**Objectives:**

To describe the case selection, technique and immediate and short-term results of balloon pulmonary valvotomy (BPV) in young infants with tetralogy of Fallot (TOF).

**Background:**

Symptomatic young infants with TOF can either undergo corrective surgery or Blalock-Taussig (BT) shunt. Corrective surgery in early infancy is associated with significant morbidity and is not a realistic option in many centers. BT shunt carries the risk of branch pulmonary artery distortion and shunt occlusion.

**Methods:**

Infants less than three months with a significant valvar pulmonary stenosis (with or without associated infundibular and annular component) and oxygen saturation ≤80% were offered BPV. The right ventricular outflow tract (RVOT) was crossed with 4F Judkin's right coronary catheter and the valve was crossed with 0.014” coronary guide wire. Serial balloon dilatations were done with over the wire coronary balloons (3-4 mm) and Mini Tyshak balloons up to a balloon annulus ratio of 2:1, depending upon the improvement in saturation and formation of annular waist.

**Results:**

Seventeen infants less than three months of age with tetralogy of Fallot (median age: 33 days, range: 10-90 days, weight: 3.47 ± 0.87 kg, pulmonary annulus Z score: -5.59 ± 1.04) including eight neonates underwent palliative BPV between May 2004 and March 2007. The mean balloon annulus ratio was 1.4 ± 0.28 and fluoroscopy time was 26.18 ± 20.2 minutes. The mean oxygen saturation increased significantly from 73 ± 7% to 90 ± 3.68% following BPV (p = 0.0001). The only major complication was RVOT perforation and pericardial tamponade in one infant. The mean follow-up period was 23 ± 12 months. Two babies developed significant desaturation requiring surgery in the six months following BPV. There was a significant increase in pulmonary annulus. The z score for the pulmonary annulus improved from -5.59 ±1.04 before BPV to - 4.31 ± 1.9 at the time of last follow-up (p = 0.018). The mean Z score of hilar right pulmonary artery (RPA) increased significantly from -1.19 ± 1.78 before BPV to 0.7 ± 0.91 after BPV (p = 0.001). The mean Z score of hilar left pulmonary artery (LPA) increased significantly from -1.28 ± 1.41 to 0.03 ± 1.29 after BPV (p = 0.005). Eight patients underwent corrective surgery.

**Conclusions:**

Balloon pulmonary valvotomy is safe and effective. It significantly improves the growth of pulmonary annulus and branch pulmonary arteries. Thus it can be considered as an interim palliative procedure for symptomatic young infants with TOF and predominant valvar pulmonary stenosis.

## INTRODUCTION

Surgical options for management of symptomatic neonates and young infants with Tetralogy of Fallot (TOF) include both complete repair and interim Blalock-Taussig (BT) shunt. Intra-cardiac repair in the neonatal period and early infancy have been achieved in limited centres with no increased risk of mortality.[1–8] But there is significant peri-operative morbidity that includes prolonged mechanical ventilation, increased inotrope requirement and end organ dysfunction.[1,3,6,7] Additional disadvantages include the need for ventriculotomy and higher risk of reoperation.[1,6–8] Due to the increased demands of postoperative care coupled with these disadvantages, many centres are reluctant to attempt primary repair of TOF in infants less than three months of age. This is particularly true for centers in the developing world where the resources are limited. The alternative to corrective operation is palliation with BT shunt in very young infants, which is still advocated.[8] The limitations of this procedure include the risk of distortion of branch pulmonary arteries in up to 15 to 20% and shunt occlusion in another 3 to 6%.[9–13] In addition, there is significant postoperative morbidity and mortality following neonatal BT shunt.[9–11,14]

BPV has been previously attempted in TOF as a palliative measure.[15–24] The right ventricular outflow tract (RVOT) obstruction in TOF is often at multiple levels viz infundibulum, valve, annulus and, main and branch pulmonary arteries. BPV can potentially offer reasonable interim palliation for infants with predominant valvar pulmonary stenosis (PS). We report our case selection, technique and, immediate and short-term results with BPV in infants less than three months of age with TOF.

## METHODS

### Patient selection

**Figure 1:**
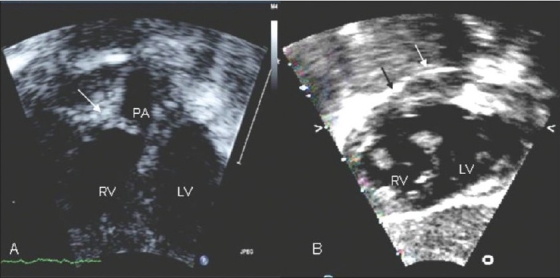
Assessing suitability for BPV by echocardiogram. A: Apical four chamber view with anterior tilt showing predominant valvar PS with doming pulmonary valve (white arrow). B: subcostal short axis view showing predominant infundibular PS (black arrow) with insignificant valvar component (white arrow). RV= right ventricle; LV= left ventricle; A= pulmonary artery

We reviewed case records of all infants aged less than three months with tetralogy of Fallot who underwent palliative BPV, BT shunt or corrective surgery between May 2004 and March 2007. All those with TOF who had saturations ≤80% were evaluated with a detailed transthoracic echocardiogram. Those with predominant valvar component of RVOT obstruction as indicated by doming pulmonary valve were selected for BPV [Figure 1]. Associated infundibular and annular narrowing were not considered as contraindications for the procedure. Infants with predominantly infundibular narrowing were excluded. Detailed informed consent was obtained from all families after explaining the available alternative treatment options.

### Cardiac catheterization

**Figure 2:**
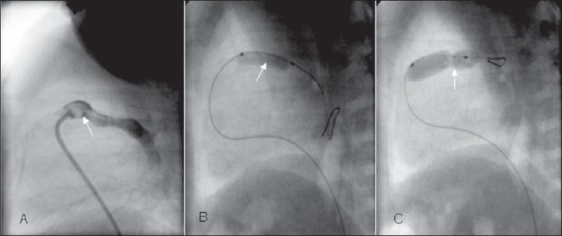
Sequence of balloon pulmonary valvotomy in the lateral view. A: Lateral view RV angiogram showing doming pulmonary valve (white arrow). B: Dilatation with coronary balloon over 0.014-guide wire showing the waist formation at pulmonary valve. C: Dilatation with an oversized Tyshak mini balloon forming a prominent waist

Procedure was done under intravenous Ketamine and Midazolam supplemented by local anesthesia. Same drugs were used for sedation during mechanical ventilation also. Adequate hydration was ensured in the peri-procedure period, including intravenous fluids for six to eight hours before and after the procedure. Catheterization laboratory was equipped with all medications to deal with a cyanotic spell. Service of anesthesia team experienced in handling cyanotic infants was available when needed. Surgical back up was available in case of any mishap. Saturations were monitored throughout the procedure using a pulse oxymeter. In addition, a short cannula was kept in the right femoral artery for monitoring blood pressure and arterial blood gases. Right ventricle was entered via the femoral vein with a 4F Judkins right coronary catheter. Right ventricular anatomy was delineated in the lateral view with a small hand injection of contrast. The catheter was then carefully maneuvered into the RVOT. In event the catheter entered the aorta (as it often did) the catheter was carefully withdrawn from the ascending aorta into the RV (guided by monitoring pressures recorded from the catheter). As soon as RV pressures were seen, the catheter was maneuvered anteriorly and to the left. The pulmonary valve was crossed in the lateral view with a 0.014” coronary guide wire with short floppy tip (3 cm), which was placed deep in the branch pulmonary artery. The selection of first balloon was arbitrary, based on assessment of angiographic anatomy by the operator. Graded dilatation was used in eight infants, of which six were initially dilated with 3 to 4 mm coronary balloons (over the wire and not monorail). It was followed by dilatation with larger Mini-Tyshak balloons [Figure 2]. In the remaining eight infants, valve was initially dilated with Mini-Tyshak balloons in the ratio of 1.2-1.6:1 and graded dilatation was not done. The end point of dilatation was individualized based on both, the improvement in saturation and formation of annular waist during dilatation. The final balloon-annulus ratio ranged from 1. 04:1 to 2:1. Balloon-annulus ratio more than 1.5:1 was used only in five patients.

### Statistical analysis

Diameters of branch pulmonary arteries and pulmonary valve (PV) annulus were expressed as Z scores. The differences between pre BPV and post BPV oxygen saturations and Z scores for the pulmonary annulus and pulmonary artery branches were compared using paired Student's T test. P value of < 0.05 was considered statistically significant.

## RESULTS

### Baseline data

Twenty-nine infants with TOF less than three months of age underwent intervention during the study period. Ten patients underwent BT shunt and two primary corrective surgery. Seventeen infants (58.6%) including one low birth weight neonate were taken up for BPV. The median age at the time of BPV was 33 days (range 10 days to 90 days). There were eight newborns. Twelve were males. Two patients had cyanotic spells. Two infants required mechanical ventilation prior to the procedure due to severe hypoxia and additional two during the procedure secondary to sedation related airway issues. Six patients had associated left pulmonary artery (LPA) origin stenosis with constricted patent ductus arteriosus (PDA) to LPA origin. Three patients had left anterior descending (LAD) artery crossing RVOT. Three patients had right aortic arch.

### Immediate results [Table 1]

Pulmonary valve dilatation could be accomplished in all except one. There was difficulty in crossing the pulmonary valve in two patients. The mean fluoroscopy time was 26.18 ± 20.2 minutes. The mean systemic oxygen saturation increased significantly from a mean of 73 ± 7 % to 90 ± 3.68% immediately following BPV (p = 0.0001). The gain in saturation ranged from 10 to 25%, the mean increase in saturation being 17 ± 6 %.

**Table 1:** Balloon pulmonary valvotomy for infants ≤ 3 months: Patient characteristics and initial outcome

Patient No.	Age in days	Weight	Baseline saturation	PA Annulus	PA annulus Z Score	Balloon/Annulus	Final saturation
1	31	3.26	80	3.9	-6.6	1.8	90
2	30	3.5	70	5	-5	1.2	92
3	31	2.89	77	4.8	-7	1.6	88
4	10	2.35	80	3	-7	2	90
5	11	1.9	65	4.2	-4.7	1.4	85
6	23	3.25	65	5.2	-4.7	1.4	85
7	12	3.1	65	4.5	-5.1	1.6	85
8*	30	2.8	78	5	-4.2	1.1	88
9	40	4.34	78	4.4	-7	1.8	90
10	56	4.96	70	5	-6	1.4	90
11	60	3.9	73	4.8	-5	1.04	92
12	85	3.57	80	4.2	-6.4	1.4	92
13	90	2.7	66.5	4.4	-5.5	1.1	96.5
14	90	3.8	78	5	-4.8	1.2	94
15	35	4.18	79	4.2	-7	1.2	95
16	90	5	60	5.8	-4.8	1.3	85
17	33	3.5	80	5.3	-4.2	ND	ND
mean	44.53	3.47	73	4.34	-5.59	1.41	90
SD	28.59	0.85	7	0.62	1.04	0.28	3.68

* Lost for follow up after 3 months, PA: Pulmonary artery, ND: BPV not done, SD: Standard deviation

### Complications

One major complication occurred. There was perforation of RVOT while attempting to cross the pulmonary valve in patient #17 [Table 2] resulting in pericardial collection and tamponade. The procedure was abandoned and pericardiocentesis done. The same patient was found to have aneurysm of the RVOT at surgery, seven months after the procedure. This finding complicated intracardiac repair. Two more patients had minor complications - transient bradycardia and ST elevation while crossing RVOT in one and moderate pulmonary regurgitation in another baby. There were no episodes of precipitation of cyanotic spells. There was no procedure-related mortality.

**Table 2:** Balloon pulmonary valvotomy for infants ≤ 3 months: Follow up results

Duration of Follow up	Outcome at most recent follow up
16 mon	Corrective operation with trans-annular patch at 7 months.
29 mon	2 1/2 years, saturating 90%, awaiting surgical correction.
15 mon	Corrective operation with trans-annular patch and LPA plasty at 6 months, disconnected LPA in the postoperative catheterization, asymptomatic.
14 mon	15 months, saturating 79%, awaiting surgical correction.
22 mon	Blalock Taussig shunt at 2months, Bidirectional Glen Shunt at 11 months (posterior pulmonary valve).
22 mon	Corrective operation with trans-annular patch at 9 months, disconnected LPA in postoperative catheterization, asymptomatic.
12 mon	Saturating 88%, awaiting surgery
3 mon	No follow up after 3 months.
45 mon	Corrective operation with trans-annular patch and LPA plasty at 5 months, balloon dilatation of LPA origin 4 months after surgery with modest result. LPA Stenting was done 3years later with good result.
19 mon	22 months, saturating 78%, awaiting surgical correction.
25 mon	Corrective operation with trans-annular patch and LPA plasty at 15 months.
45 mon	Blalock-Taussig shunt at 6 months (coronary crossing right ventricular outflow tract), corrective operation with pericardial conduit (double barrel repair) at 2 years.
25 mon	2 1/4 years, saturating 78%, conservative management as child has severe global developmental delay.
11 mon	Intracardiac repair with pericardial conduit (dual left anterior descending coronary artery) with LPA plasty at 14 months. Died of low cardiac output in the 4th postoperative week.
45 mon	Corrective operation with trans-annular patch and LPA plasty at 7 months.
15 mon	Required BT shunt at 1 1/2 years due to low saturation coronary crossing RVOT and diffusely small LPA.
25 mon	Bi-directional Glenn shunt at 8 months (RVOT aneurysm detected at surgery).

### Follow up [Table 2]

Mean follow-up period was 23±12 months. Two babies developed significant desaturation requiring surgery in the six months following BPV. One of them underwent BT shunt at two months of age, 1.5 months after BPV and second infant underwent corrective surgery four months after BPV at the age of five months. One patient was lost to follow-up after three months and only limited information by telephonic enquiry could be obtained after that. The mean saturation at three months follow-up was 83 ± 9%. Two more patients required interim BT shunt six and 15 months after BPV as LAD crossing RVOT excluded early complete repair.

### Corrective surgery

Eight patients have already undergone elective corrective surgery (including the one who had BT shunt at six months of age) at mean age and weight of 9 ± 4months and 6.7 ± 1.15 kg respectively. There was one surgical mortality from refractory low cardiac output state with multi-organ failure in the early postoperative period after corrective surgery. In addition, two patients had to undergo bi-directional Glenn shunt (BDGS) instead of TOF repair due to unexpected anatomical issues on table: relatively posterior pulmonary valve in one and aneurysm of RVOT, as stated earlier, in another. In both the cases, these details were not identified in the preoperative assessment. Both were suitable for two-ventricle repair and BDGS was done as an interim palliation. First patient would have required conduit, which was not readily available, and second case required detailed surgical planning which could not be done beforehand. All of the patients who underwent corrective surgery required either transannular patch (n=6) or double barrel repair of RVOT (n=2). Six required LPA plasty too.

### Growth of the pulmonary annulus and branch pulmonary arteries

There was a statistically significant increase in pulmonary annulus Z score from -5.59 ±1.04 before BPV to - 4.31 ± 1.9 on follow-up after BPV (p = 0.018). The mean Z score of hilar RPA increased significantly from -1.19 ± 1.78 before BPV to 0.7 ± 0.91 after BPV (p = 0.001). The mean Z score of hilar LPA increased significantly from -1.28 ± 1.41 to 0.03 ± 1.29 after BPV (p = 0.005).

## DISCUSSION

Since the 1980s there have been several case series of palliative BPV in TOF with most reporting favorable outcome.[15–24] Most of these earlier series included a wide age group of patients. A number of patients reported could perhaps undergo primary correction in the current era. Our case series is different in that only infants less than three months with SaO2 ≤80% and having predominantly valvar PS were considered for BPV. We selected this particular age group because repair in early infancy is not routinely practiced at our centre and most infants with symptomatic TOF would have otherwise required an interim BT shunt. We could palliate more than 50% infants less than three months with TOF who required intervention during the study period.

There were many other series where neonates and young infants were a part of the cohort,[15,17–19–21,24] reporting variable outcome. Most of these series reported statistically significant improvement in saturation, similar to our data.[15,17–21,24] The variable degree of improvement in saturation between patients maybe due to varying severity of obstruction at different levels of RVOT. But, sustained improvement in saturation so as to avoid further palliation was not achieved by all. In general, most of the series reported that further palliative procedures could be avoided in about 75 to 100% of patients.[15,17,19–21] But, there are reports of poor outcome too. Piechaud *et al*,[18] reported that only 47.5% patients in their series had stable successful procedures. Recently, Wu *et al*,[24] reported that 45.5% required BT shunt in a median of 11 days after BPV. Both the series had reported poor outcome particularly in young infants and neonates. Our series differs from others in having a high success rate of 87.5% in neonates and young infants with only two patients requiring surgery within six months of initially successful BPV. This result underscores the significance of appropriate case selection and attention to procedural details. The mean pulmonary annulus in neonatal and infant BPV in previous reports ranges from a Z score of -4 to -4.8,[19,21] which is larger compared to -5.59 ± 1.04 in our series. The mean balloon annulus ratio of 1.4 ± 0.28 in our series is similar to that reported by others.[19,21] However, there was no significant pulmonary regurgitation except in one who developed moderate pulmonary regurgitation.

It has been suggested that patients suffering hypoxic spells are not good candidates for dilatation.[16,18,19] The presence of recurrent cyanotic spells was the most important predictor of failure in one series.[24] However, good outcome after dilatation has been reported in patients with cyanotic spells too.[21] We had only two infants with cyanotic spells, which is too small a number to comment. There are also reports that BPV can precipitate spells or transient reduction in saturation.[19,21] We did not experience such events probably due to multiple factors including appropriate patient selection, adequate hydration and use of ketamine, which increased systemic vascular resistance.

Major procedural complication reported is perforation and tamponade, as in our series.[18,19] Pseudoaneurysms, healed posterior tear of pulmonary artery and other evidences of structural damage have been reported later during corrective surgery.[25–27] However, most of the series including ours did not report any procedure-related mortality.[15,17,19,21,24]

Like many of the earlier series,[19–22] we observed significant growth of the branch pulmonary arteries and pulmonary annulus following BPV. Growth can be explained by the symmetric increase in antegrade flow after BPV. However, no series including ours have a control group to reinforce this argument. Many studies have looked in to the efficacy of BPV in reducing the need for transannular patching.[19–21] Significant growth of pulmonary valve annulus has not translated in decreasing the use of a transannular patch in all. Sluysmans *et al*,[19] has reported an incidence of transannular patching in 31% among the 16 patients who underwent corrective surgery. This represented 40% reduction in the need for transannular patch. However Godart *et al*,[21] has reported a 43% requirement of transannular patching despite significant growth of pulmonary annulus. The mean pulmonary annuls at follow-up was similar in both these series (-2.7 and -2.5 respectively). Therefore it is likely that the difference in the need for tranannular patch may reflect surgical preference, as supported by data from the recent series where pulmonary annulus preservation was possible even with annulus Z score of - 4 in up to 80% of patients.[28–30] All the eight patients who underwent intra-cardiac repair in our series required transannular patch or double barrel repair. This is expected as the pulmonary annuls before BPV was smaller in our series and though it increased significantly, the mean Z score of - 4.31 ± 1.9 on follow-up is too small to avoid transannular patch.

### Limitations

The major limitation of the present series is the relatively small number of patients. In addition, the data is retrospective and we did not have a control group.

## CONCLUSIONS

Balloon pulmonary valvotomy can be considered as an interim palliative procedure for young infants with TOF and predominant valvar pulmonary stenosis. Appropriate case selection and attention to procedural details are essential for success. The pulmonary annulus and branch pulmonary arteries show a significant increase in size after BPV. Thus in a selected group of patients BPV is a viable alternative to BT shunt.
